# Late-Onset Ornithine Transcarbamylase Deficiency and Variable Phenotypes in Vietnamese Females With *OTC* Mutations

**DOI:** 10.3389/fped.2020.00321

**Published:** 2020-07-23

**Authors:** Huy-Hoang Nguyen, Ngoc Khanh Nguyen, Chi Dung Vu, Thi Thu Huong Nguyen, Ngoc-Lan Nguyen

**Affiliations:** ^1^Institute of Genome Research, Vietnam Academy of Science and Technology (VAST), Hanoi, Vietnam; ^2^Department of Endocrinology, Metabolism and Genetic, Center for Rare Diseases and Newborn Screening, Vietnam National Hospital of Pediatrics, Hanoi, Vietnam; ^3^Graduate University of Science and Technology, Vietnam Academy of Science and Technology (VAST), Hanoi, Vietnam

**Keywords:** ornithine transcarbamylase deficiency (OTCD), Vietnamese females, heterozygous females, c.365A>T, IVS7+1G>A

## Abstract

**Background:** Ornithine transcarbamylase deficiency (OTCD) is an X- linked recessive disorder and the most common error of the urea cycle, caused by the mutations in the *OTC* gene. Due to X-inactivation, 15–20% of female carriers present symptoms of OTCD at late onset. Early diagnosis of OTCD by molecular analysis in females is highly desirable. The aim of the study was to identify the mutations in two unrelated Vietnamese girls suspected with OTCD and the carriers in their families for definitive diagnosis and proper counseling.

**Case Presentation:** Two patients presented with an acute encephalopathy at the first admission. Biochemical tests revealed hyperammonemia, hyperlactatemia, elevated glutamine level, elevated transaminase, elevated urinary orotic and uracil acid levels, and disorder of prothrombin time. Brain magnetic resonance imaging indicated cerebral edema. Based on the clinical and laboratory results, the two patients were diagnosed with urea cycle disorders. Therefore, the two patients were managed by stopping feeding, with infused glucose, l-carnitine, l-arginine, and sodium benzoate, and with hemofiltration. The two patients were alert and recovered with normal blood ammonia levels after 72 h of treatment. The family history of patient 1 showed that her brother died at 4 days of age due to a coma and dyspnea, while her parents were asymptomatic. Variable phenotypes were observed in three generations of the patient 2's family, including asymptomatic (mother), affected female adults dying at the first symptom (grandmother and aunt), and affected males dying in the first week of life (uncle, cousin, and siblings). Whole-exome sequencing showed two mutations in the *OTC* gene, including one novel missense mutation, c.365A>T, in the patient 1 and one previously reported splicing mutation, c.717+1G>A, in the patient 2. The two mutations are evaluated as likely pathogenic and pathogenic, respectively, according to the recommendations of the American College of Medical Genetics and Genomics (ACMG). Genetic analyses in the families indicated the mothers were heterozygous.

**Conclusion:** Clinical, biochemical, and molecular findings accurately diagnosed the two patients with late-onset OTCD. Our results explained the genetic causes and proposed the risk in the patients' families, which could be useful for genetic counseling and monitoring in prenatal diagnosis.

## Introduction

Ornithine transcarbamylase is a pivotal enzyme involved in the urea cycle. It is a mitochondrial enzyme and is responsible for the synthesis of citrulline from carbamyl phosphate and ornithine (1). Ornithine transcarbamylase deficiency (OTCD, OMIM # 311250) is the most defect of the urea cycle, with an estimated prevalence range from 1 in 14,000 (2) to 1 in 80,000 people (3). OTCD is an X-linked recessive genetic disorder and causes heterogeneous clinical symptoms. Hemizygous males have a spectrum of severity ranging from complete OTCD, which presents at the neonatal onset with acute hyperammonemia, to partial OTCD, which presents mild symptoms at late onset (4). Due to a skewed X-chromosome inactivation, leading to the wild-type allele of the two X-chromosome allele in females being randomly inactivated, ~15% of heterozygous females with OTCD show the mild late-onset phenotypes (5). OTCD is biochemically characterized by profound plasma hyperammonemia, reduced level of citrulline, and elevated glutamine and urinary orotic acid. The clinical manifestations include irregular episodes of vomiting, progressive lethargy, and irritability. The disorder may rapidly progress to include coma, confusion, seizures, hypotonia, hepatomegaly, and edema. Patients may also relapse into clinical signs like protein aversion, headaches, and behavioral changes.

OTCD is caused by mutations of the *OTC* gene, which is located on the short arm of the X chromosome (Xp21.1) and encodes mature OTC proteins composed of 322 amino acids (6). To date, over 500 mutations have been recorded in the *OTC* gene (7). Single base substitutions account for about 70–84% of *OTC* mutations, small-fragment deletions or insertions represent about 12%, and 4% is represented by large-fragment deletions (8). The disease severity depends on the activity of OTC, which is affected by the type and the site of mutations (9, 10). The large deletions, frameshift, and nonsense mutations evidently cause a complete loss of OTC activity, resulting in a neonatal form of the OTCD, while missense and splicing mutations lead to a partial loss of OTC activity, resulting in both of neonatal and late onset of the disease (11). However, missense mutations appearing in the active sites resulted in a nearly complete loss of OTC activity, leading to a severe OTCD (12).

In this study, we report the clinical features and molecular analyses of two Vietnamese girls suspected with OTCD and their families.

## Case Presentation

### Clinical Presentation

Patient 1: A 12-month-old girl of non-consanguineous Vietnamese parents presented with fever, cough, anorexia, and lethargy for 2 days, then was referred and managed with a diagnosis of encephalopathy at the local hospital for 1 day. When her consciousness worsened, she was transferred to our emergency. On admission, she presented with mechanical ventilation, shock condition, deep coma, and dilation of both pupils to 4 mm with weak light reaction (Table 1). The biochemical investigation revealed hyperammonemia, elevated transaminase, hyperlactatemia, elevated blood glutamine, elevated blood lysine, elevated phenylalanine, elevated urinary orotic and uracil acid levels, and disorder of prothrombin time. The results of brain magnetic resonance imaging (MRI) revealed cerebral edema and abnormal T1W. She was managed by stopping feeding, with infused glucose (10 mg kg^−1^ min^−1^), L-carnitine (100 mg kg^−1^ day^−1^), and L-arginine (500 mg kg^−1^ day^−1^), and with hemofiltration. After 72 h, she was alert and recovered, with normal blood ammonia level. For long-term outcome, at the age of 3, she had a development delay, with a development quotient (DQ) around 50%, and had five recurrent episodes of hyperammonemia. She was the first child and was born at 38 weeks of gestation, with a birth weight of 2.8 kg and normal development. The family history showed that her brother died at 4 days of age due to a coma and dyspnea. Her parents were reported asymptomatic.

**Table 1:** Clinical and molecular analyses of two Vietnamese female patients with OTCD.

	**Patient 1**	**Patient 2**
**CLINICAL SYMPTOMS**		
Age of diagnosis	12 months	24 months
Initial symptoms	Fever, cough, anorexia, and lethargy.	Vomiting, anorexia, and lethargy.
Presenting symptoms	Mechanical ventilation, shock condition, deep coma, both of dilated pupils at 4 mm with weak light reaction.	Coma, no paralysis, no convulsion
Family history	Brother died at 4 days of age due to a coma and dyspnea. Parents were asymptomatic.	Uncle, two brothers, and cousin died after birth due to coma and dyspnea. Grandmother died at the age of 35 by a stroke. Aunt died at the age of 21 after delivery due to a coma. Parents and elder sister were asymptomatic.
Initial ammonia levels (μg/dL) (Normal: <50)	1,100	259
AST levels (UI L^−1^) (Normal: 10–40)	148	53
ALT levels (UI L^−1^) (Normal: 7–59)	119	69
INR	3.45	3.03
Glutamine levels (μmol L^−1^) (Normal: 530 ± 81)	1,490	579
Lactatemia levels (mmol L^−1^) (Normal: 1.1–2.3)	8.3	4.75
Urinary orotic levels	Elevated	Elevated
Uracil acid levels	Elevated	Elevated
Lysine levels (μmol L^−1^) (Normal: 48–284)	430	135
Phenylalanine levels (μmol L^−1^) (Normal: 26–91)	116.8	31.7
Brain MRI/CT scanner Findings	Cerebral edema and abnormal T1W	Cerebral edema
Management	Stop feeding, glucose infusion (10 mg kg^−1^ min^−1^), l-carnitine (100 mg kg^−1^ day^−1^), and l-arginine (500 mg kg^−1^ day^−1^), and hemofiltration	Stop feeding, glucose infusion (10 mg kg^−1^ min^−1^), l-carnitine (100 mg kg^−1^ day^−1^), l-arginine (500 mg kg^−1^ day^−1^), and sodium benzoate (500 mg kg^−1^ day^−1^)
**LONG-TERM OUTCOME**		
Age	3	4
Development	Delay (DQ = 50%)	Delay (DQ = 50%)
Recurrent episodes of hyperammonemia	5	10
**MOLECULAR ANALYSES**		
Nucleotide change	c.365A>T	c.717+1G>A
Position in the *OTC* gene	Exon 4	Intron 7
Zygosity	Heterozygous	Heterozygous
Maternal status	Carrier	Carrier
Effect	Missense	Donor splice site error
Amino acid change	p.Glu122Val	–
SIFT (score/prediction)	0 (Deleterious)	–
PolyPhen-2 (score/prediction)	1 (Damaging)	–
PANTHER (score/prediction)	4200 (Probably damaging)	–
FATHMM (score/prediction)	−6.61 (Damaging)	–
SNPs&GO (score/prediction)	RI 9 (Disease)	–
Pmut (score/prediction)	0.93(94%) (Disease)	–
Align-GVGD (score/prediction)	C65 (Pathogenic)	–
SNAP2 (score/prediction)	88 (Effect)	–
Mutation assessor (score/prediction)	4.24 (High impact)	–
PROVEAN (score/prediction)	−6.625 (Deleterious)	–
CADD (score/prediction)	34 (Deleterious)	33 (Deleterious)
Mutation Taster (score/prediction)	0.9999 (Disease causing)	1 (Disease causing)
MaxEntScan	–	0.94 (Mutant) vs. 9.12 (Wild type) (−89.69% variation)
Splice finder	–	60.43 (Mutant) vs. 87.26 (Wild type) (−30.75% variation)
Frequency (1,000 genomes, gnomAD)	0	0
ClinVar	–	Pathogenic (RCV000083542.1)
ACMG classification	Likely pathogenic	Pathogenic
Human gene mutation database	Novel	HGMD ID CS003075

Patient 2: A 24-month-old Vietnamese girl presented with vomiting, anorexia, and lethargy for 2 days and then was admitted and managed with a diagnosis of encephalopathy at the province hospital for 1 day. When her consciousness worsened, she was transferred to our emergency. On admission, she presented with a coma, no paralysis, and no convulsion (Table 1). The biochemical analyses revealed mild hyperammonemia, elevated alanine aminotransaminase and aspartate aminotransaminase, high glutamine level, elevated lactatemia, elevated urinary orotic and uracil acid levels, normal lysine and phenylalanine levels, and disorder of prothrombin time. The results of the MRI revealed cerebral edema. She was managed by stopping feeding and infused glucose (10 mg kg^−1^ min^−1^), L-carnitine (100 mg kg^−1^ day^−1^), L-arginine (500 mg kg^−1^ day^−1^), and sodium benzoate (500 mg kg^−1^ day^−1^). She was alert and recovered with normal blood ammonia level after 72 h of treatment. At the age of 4, she had a development delay (DQ = 50%) and 10 recurrent episodes of hyperammonemia. Hemofiltration was performed to reduce the blood ammonia level. She was the fourth child of non-consanguineous Vietnamese parents. Her birth weight was 2.9 kg at 38 weeks of gestation. She had normal development at the time of birth.

**Figure 1:**
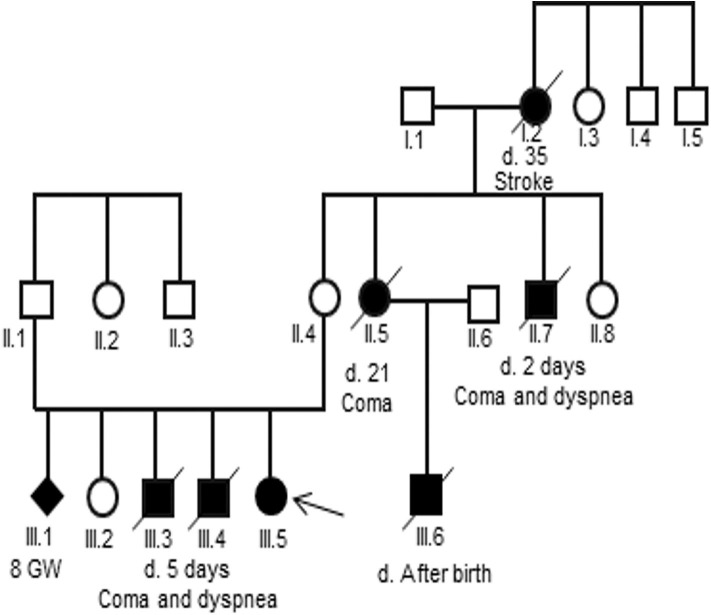
Pedigree of the three-generation family of the patient 2. Generations are shown as I to III. Males are indicated by squares, females by circles, affected members by a shaded black square or circles and the proband by an arrow. Shaded black square with slash indicates death.

Analysis of the pedigree disclosed a marked family history of an X-linked maternal inheritance in the family of patient 2 (Figure 1). Her uncle and two her elder brothers died at 2 and 5 days, respectively, after birth due to a coma and dyspnea. Her cousin, son of her aunt, also died after birth. However, a skewed X-inactivation may lead to variable clinical features in the females in this family. In detail, her grandmother died at the age of 35 of a stroke, her aunt died at the age of 21 after delivery due to a coma, while her mother and her sister were asymptomatic.

### Molecular Investigation

Genomic DNA samples were extracted from peripheral blood samples of the two patients and from avaiable members of their families using QIAamp DNA blood mini kit (Qiagen, Germany), following the manufacturer's protocol. Whole-exome sequencing (WES) was applied to detect variants in the two patients. The DNA libraries were prepared with an Agilent SureSelect Target Enrichment Kit (Agilent Technologies, CA, USA) according to the manufacture's protocol. The exome libraries were captured and amplified using an Agilent SureSelect Human All exon v6 Kit (Agilent Technologies, CA, USA), then sequenced with the Illumina platform sequencer (Illumina, CA, USA).

The sequencing reads were mapped to the GRCh37/hg19 reference human genome using the Burrows–Wheeler Aligner tool v0.7.12 (13). The duplicate reads were marked and removed using Picard tool v1.130 (http://broadinstitute.github.io/picard/). Variant calling and annotation were performed using the Genome Analysis Toolkit v3.4.0 (14) and SnpEff v4.1g (http://snpeff.sourceforge.net/SnpEff.html), respectively. The population frequency of the variants was estimated by comparing with the Exome Sequencing Project (https://evs.gs.washington.edu/EVS/) and 1,000 Genome (http://browser.1000genomes.org). Variants with a minor allele frequency > 0.01 were excluded. The pathogenicity of the variants was evaluated based on the information in the ClinVar database (https://www.ncbi.nlm.nih.gov/clinvar) and *in silico* analyses. Sorting Intolerant from Tolerant (SIFT) (15) and Polymorphism Phenotyping v2 (PolyPhen-2) (16) were applied to predict the effects of missense variants. The mutation Taster program (17) and Combined Annotation Dependent Depletion (CADD) (18) were used for the pathogenic prediction of the candidate variants. Human Splicing Finder (HSF) software (19) and MaxEntScan program (http://hollywood.mit.edu/cgi-bin/Xmaxentscan_scoreseq.pl) were applied to estimate the effect of the splice site candidate variant on the splice site strengths and the presence of putative splicing regulatory elements. The pathogenicity of the missense candidate variant was further evaluated using the SNAP2 (https://www.rostlab.org/services/snap/), Align-GVGD (http://agvgd.hci.utah.edu/agvgd_input.php), PANTHER (http://www.pantherdb.org/tools/hmmScoreForm.jsp), SNPs & GO (https://snps.biofold.org/snps-and-go/snps-and-go.html), protein variation effect analyzer (PROVEAN) (20), FATHMM (http://fathmm.biocompute.org.uk/inherited.html), mutation assessor (http://mutationassessor.org/r3/), and Prediction of Pathological Mutations on proteins (Pmut) (http://mmb.irbbarcelona.org/PMut/analyses/new). The read depth of the candidate variants was checked using the Intergrative Genomics Viewer (IGV 2.6.3) (21).

**Figure 2:**
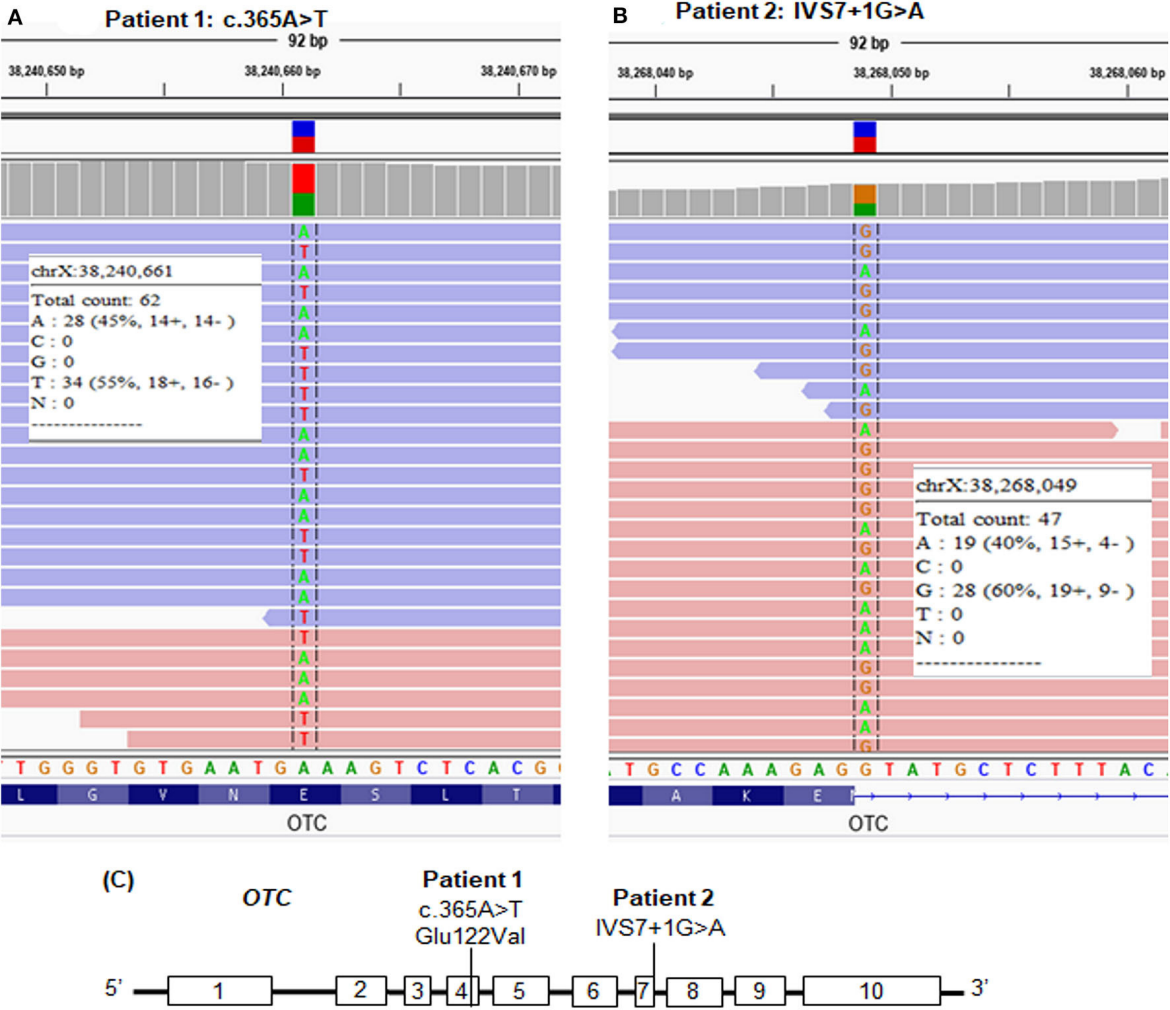
Whole-exome sequencing (WES) paired-end reads are loaded in the IGV genome browser and are shown for each patient. **(A)** The heterozygous mutation, c.365A>T, in patient 1. **(B)** The heterozygous mutation, IVS7+1G>A, in patient 2. **(C)** Location of the point mutations in the *OTC* gene.

**Figure 3:**
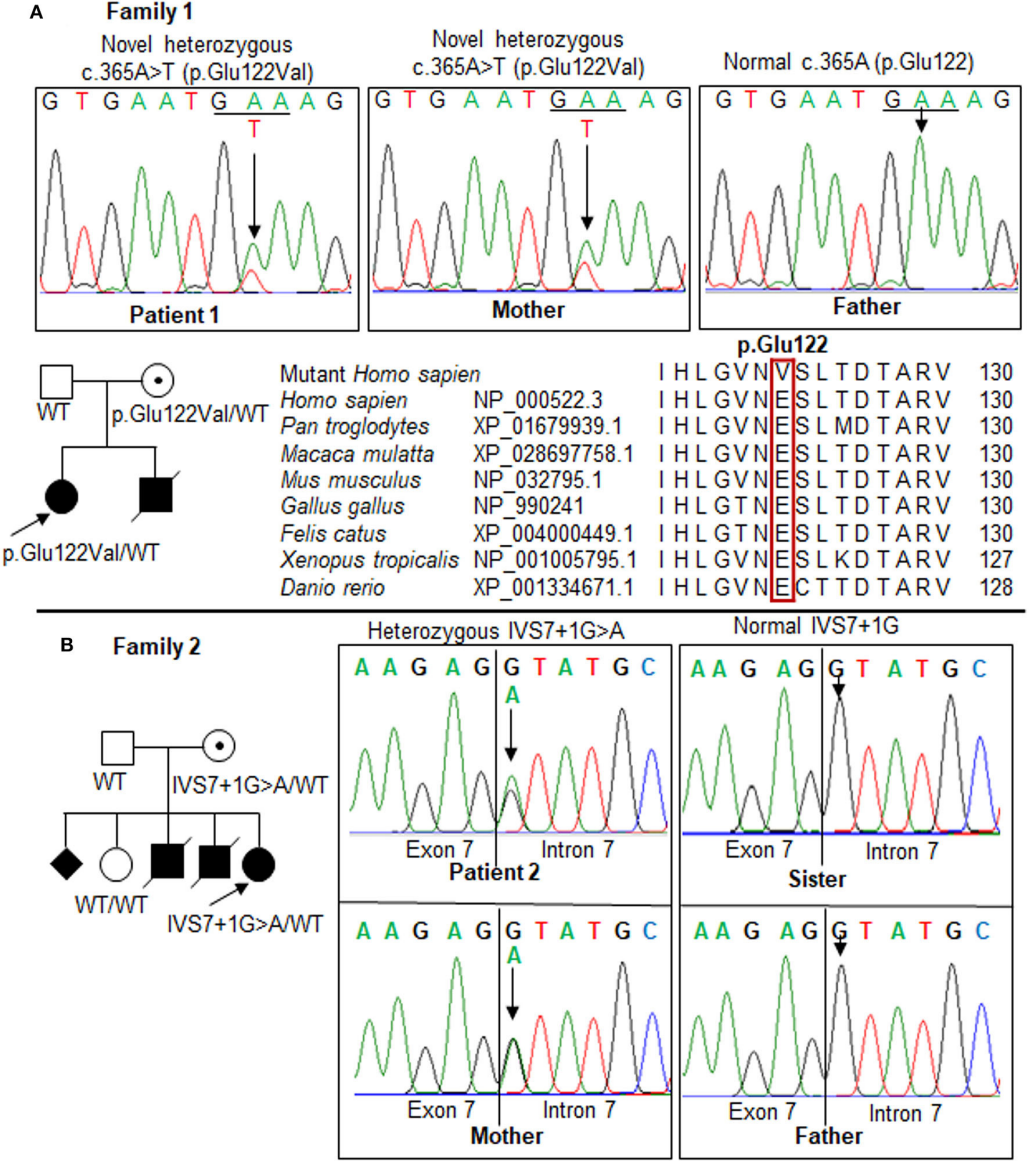
Mutations in the *OTC* gene in the two families. Pedigrees of the two families show the affected (*solid*), unaffected (*open*), and asymptomatic carrier (*circle with dot*) members. *Squares*, males; *circles*, females; *slashed symbol*, deceased. The genotype is shown *underneath each symbol*; WT, wild-type. **(A)** Patient 1 and her mother carried a novel heterozygous missense mutation, c.365A>T (p.Glu122Val), in exon 4. Multiple-sequence alignment of the amino acid sequences of *OTC* from different species revealed a complete conservation of the p.Glu122 residue across species (*red box*). **(B)** Identification of a mutation, IVS7+1G>A, in the *OTC* gene in the family of patient 2. The results showed a maternal carrier, while the father and sister were normal. The GenBank reference sequences of the human *OTC* gene are NM_000531.6 for the mRNA sequence and NC_000023.11 for the exon-intron boundary.

We screened the candidate genes associated with hyperammonemia and elevated glutamine and selected the variants which had a frequency ≤ 1% and were evaluated as disease causing by *in silico* analyses. We found a novel missense mutation, c.365A>T, in exon 4 of the *OTC* gene in patient 1 (Table 1 and Figures 2A,C) and a reported splice site mutation, IVS7+1G>A, in intron 7 of the *OTC* gene in patient 2 (Table 1 and Figures 2B,C). The two mutations were heterozygous. The read depth for variation c.365A>T was 62, with the mutant allele T accounting for 55%. The read depth for variation IVS7+1G>A was 47, with the mutant allele A accounting for 40%. These mutations were confirmed by Sanger sequencing (Figure 3). Such molecular investigation supports a definitive diagnosis for the two patients with OTCD.

In patient 1, the replacement of A to T at position 365 leads to switch glutamate (Glu) at residue 122 to valine (Val). Sanger sequencing revealed the mother carries the mutation, but the father does not. The proband and mother were heterozygous for this mutation. The p.Glu122Val mutation was predicted to be “disease causing” by the Mutation Taster (Supplementary Table 1), SNP&GO, and PMut analyses; “probably damaging” by the PolyPhen-2, PANTHER, and FATHMM analyses, “deleterious” by the SIFT, PROVEAN, and CADD analyses; “high effect” by the SNAP2 and Mutation assessor analyses; and “pathogenic” by the Align-GVGD analysis (Table 1). The amino acid sequences of OTC from human and other species were compared to identify the conservation at the mutant position. A glutamate residue at the position 122 was completely conservative among distinct species (Figure 3A). The alternation of Glu to Val at the conservative position may lead to a change in the protein structure, which may affect the enzyme stability or activity. The c.365A>T has not been previously reported in the 1,000 Genomes Project, Exome Variant Project, Human Genome Mutation Database, ClinVar database, Genome Aggregation Database, and in-house WES database (*n* = 54); therefore, c.365A>T is a novel mutation in the *OTC* gene. This variant is classified as likely pathogenic according to the ACMG criteria (Supplementary Table 2).

In patient 2, a heterozygous splice site mutation, c.717+1G>A (IVS7+1G>A), was identified at the first nucleotide of intron 7 of the *OTC* gene. This mutation was previously reported as a pathogenic variant in ClinVar (RCV000083542.1, rs66500027) and predicted as a disease-causing variant in the Mutation Taster analysis (Supplementary Table 1) and a deleterious variant in the CADD analysis (Table 1). This alternation disrupted a highly conserved GT dinucleotide sequence at the splice donor site. Sanger sequencing showed that the IVS7+1G>A mutation was heterozygous in patient 2 (Figure 3B). IVS7+1G>A may affect normal splicing of exon 7. The G>A substitution at the first nucleotide in intron 7 resulted in a lower maximum entropy (MaxENT) score than wild-type (0.94 vs. 9.12, respectively). Human Splicing Finder analysis indicated that the IVS7+1G>A mutation could cause an alteration of the wild-type donor site, most probably affecting splicing (−30.75% variation). Inherited genetic analysis revealed the carrier as the mother, while the father and sister were not. The c.717+1G>A variant is considered as pathogenic according to the ACMG recommendations (Supplementary Table 2).

## Discussion

Our patients presented the typical signs and symptoms of urea cycle disorders such as anorexia, lethargy, vomiting, coma, bilaterally dilated pupils, cerebral edema, development delay, hyperammonemia, elevated glutamine, elevated urinary orotic acid, and disorder of prothrombin time (7). In addition, neonatal death of the males and the variable phenotypes in the females in the patients' families support an X-linked maternal inheritance. In the urea cycle disorders, only OTCD is inherited in an X-linked manner; therefore, the two patients were suspected with OTCD. Molecular analysis is the first choice to obtaining an accurate diagnosis of these patients (22). In this study, by using WES, we identified one missense mutation, c.365A>T, in patient 1 and one splicing mutation, IVS7+1G>A, in patient 2. Such results established a definitive diagnosis of the two patients with OTCD. The onset of symptoms of our patients was from 12 to 24 months old, respectively, therefore, we classified them to late onset, as suggested by Hediger et al. (23).

Hyperammonemia and high brain glutamine can affect the central and peripheral nervous system (24). The early neurological effects like anorexia and vomiting and progressive central nervous system dysfunction like lethargy and coma occurred in our patients. Neurological damage is further confirmed in patients 1 and 2 by cerebral edema, which was also reported in OTCD patients in the studies of Chongsrisawat et al. (25) and Hershman et al. (26). Acute hyperammonemia may induce liver function impairment. The most frequently liver-related complication in OTCD is acute liver failure with coagulopathy (27). Classification of the acute liver injury in patients 1 and 2 is liver dysfunction due to INR>2, as described by Gallagher et al. (28). A total of 75, 50, and 40% of neonatal males and patients with moderate and mild OTCD, respectively, showed liver injury, but only 9% of asymptomatic patients showed liver dysfunction in the study of Gallagher et al. (28). In the study of Laemmle et al. (29), acute liver failure was also reported in nine of nine male patients, but 6 in 15 symptomatic females and none in five asymptomatic females with OTCD; therefore, the percentage of symptomatic females showing liver injury ranged from 40 to 50%. In contrast, two of the two (100%) symptomatic female showed liver dysfunction in our study. The number patients in this study is small. This is a weakness of our study.

A novel heterozygous missense mutation, c.365A>T (p.Glu122Val), was predicted as causing disease in patient 1 based on the 12 computational algorithms (Mutation Taster, CADD, PolyPhen-2, PANTHER, FATHMM, SNPs&GO, PMut, Aling-GVGD, SNAP2, Mutation Assessor, PROVEAN, and SIFT). The switching of a negative charge hydrophilic glutamate to a hydrophobic valine in the polypeptide chain might have caused the clumping together due to staying away from the water, resulting in impaired folding of the protein. Residue Glu122 is involved in the subunit interactions with residue Arg92, which maintains the stabilizing of the tetrahedral carbon in the loop that connects β strand 3 with α-helix 3 (30, 31). Moreover, the mutation occurred in the highly evolutionary conserved position; hence, the replacement of Glu 122 to Val potentially impacted the normal configurations of the protein structure.

To the best our knowledge, a total of 12 pathogenic missense mutations have been reported in exon 4 of the *OTC* gene (Supplementary Table 3). These mutations are amorphic, which causes the loss of function of OTC. The majority of which (9/12, 75%) presents at late onset in either males or females. Only three of which (25%) appeared at neonatal onset in male. In the patient 1's family, the brother of the proband died due to a coma and dyspnea; therefore, we suspect that the brother also carried the mutation p.Glu122Val. Although the mother of the proband is a carrier of the heterozygous Glu122Val mutation, she had no apparent clinical symptoms until now. This could be explained by the random X- chromosome inactivation or the severity of the mutation (25, 32). At residue 122, the replacement of Glu with Gly was previously identified in late-onset OTCD in a Chinese family of Han nationality (33) and in a Spanish male (12). The alternation p.Glu122Gly was predicted to cause partial deficiency by eliminating the intersubunit connections between the side-chain carboxylate of Glu122 and the guanidinium group of Arg92 (12). Glycine is also a hydrophobic like valine. In this manner, the mutation p.Glu122Val may reduce enzyme activity, resulting OTCD in patient 1. However, p.Glu122Gly resulted in a late-onset of OTCD in 13-month-old Spanish male (12), while p.Glu122Val may cause a neonatal-onset of OTCD in patient 1's brother.

A heterozygous splice site mutation, IVS7+1G>A, appeared on the first nucleotide of intron 7 in patient 2. The alternation of within GT dinucleotide at the splice donor sequence may induce exon skipping, partial exon loss, intron retention, and cryptic donor splice site activation, in which exon skipping is common (34). The size of exon 7 and intron 7 is short (54 and 80 nucleotides, respectively); hence, exon 7 skipping may occur, leading to a truncated mRNA chain. Splice site mutations accounted for about 8% of all disease-causing mutations in late-onset OTCD (35). The mutation IVS7+1G>A was reported in a neonatal-onset male with OTCD (36). Our case was the second to undergo the mutation IVS7+1G>A. Except IVS2+1G>A which cause the late-onset in males (7, 37), most of +1G>A mutations cause the neonatal-onset in males and the late-onset in females (10), in which the neonatal-onset disease was more prevalent than the late phenotypes. This trend also observed in the family of patient 2 with variable phenotypes in three generations, including asymptomatic (mother), affected female adults dying at the first symptom (grandmother and aunt), and affected males dying in the first week of life (uncle, cousin, and siblings). We suggested they might also have OTCD, but misdiagnosed.

Approximately 80% of heterozygous females are mostly healthy; however, 20% of those are symptomatic hyperammonemia, in which adult heterozygous females can have hyperammonemia episodes after changing to high protein intake, acute illness, trauma, pregnancy, delivery, or corticosteroid use (38). Therefore, the mothers of patients 1 and 2 are at high risk of OTCD and need to have routine medical monitoring for proper management. Torkzaban et al. (39) summarized that, when the diagnosis of maternal OTCD is known prior to pregnancy, this reduces the maternal and neonatal morbidity and mortality compared to when the diagnosis is obtained during pregnancy. In detail, 31% (5/16) of undiagnosed prior to pregnancy maternal OTCD died, while no one (0/20) was observed in the diagnosed prior to pregnancy group. In consequence, the detection of the carrier status in the mothers of patient 1 and 2 may significantly improve the maternal outcomes of their subsequent pregnancies, as suggested by Torkzaban et al. (39). Moreover, any new offspring from the carriers should also undertake genetic testing to determine the status of their OTC alleles for appropriate genetic counseling and early medical intervention.

Genetic testing identified the mutations in the *OTC* gene in the patients, leading to an accurate diagnosis of the patients with OTCD. Based on these findings, the patients obtained an appropriate management, such as treatment with the drugs for lowering of blood ammoniac level like sodium benzoate, arginine, or citrulline, intake of a protein-restricted diet, and a plan for liver transplantation in severe conditions. The positive genetic results are also useful for genetic counseling. Genetic testing helped to identify the asymptomatic female carriers who might be at risk of hyperammonemia in the future. In addition, genetic testing provided an answer about the family history of the patient 2 and useful information when parents are planning for a new child. Therefore, genetic testing, especially next generation sequencing, is an established test method in clinical managements (40, 41). Integrating genetic and clinical data enhances the care quality for the patients as well as their families (40–43).

## Conclusions

In conclusion, two heterozygous mutations, c.365A>T and IVS7+1G>A, in the *OTC* gene were detected for the first time in two Vietnamese girls with OTCD. Females carrying these mutations should be considered at risk for hyperammonemia episodes and need appropriate counseling and surveillance. Males in these families need emergency treatment plan after delivery. Further studies using animal models are required for the analysis of the detail effect of the mutations with OTCD.

## Data Availability Statement

The original contributions presented in the study are included in the article/Supplementary Material, further inquiries can be directed to the corresponding author/s.

## Ethics Statement

This study was conducted in accordance with the Declaration of Helsinki, and the protocol was approved by the Ethics Committee of the Institute of Genome Research (No. 18/QD-NCHG, Institute of Genome Research Institutional Review Board, Hanoi, Vietnam). For minors, written informed consent for sample collection and genetic analyses were signed by their parents. Written informed consent was obtained from the minor(s)' parents, for the publication of any potentially identifiable images or data included in this article.

## Author Contributions

H-HN and NK conceptualized, designed the study, and wrote and reviewed the manuscript. CD provided patients' clinical information and reviewed the manuscript. N-LN performed whole-exome sequencing, analyzed data, and wrote and reviewed the manuscript. TT carried out Sanger sequencing. All authors contributed to the article and approved the submitted version.

## Conflict of Interest

The authors declare that the research was conducted in the absence of any commercial or financial relationships that could be construed as a potential conflict of interest.
