# Supplementary material for: Late-Onset Ornithine Transcarbamylase Deficiency and Variable Phenotypes in Vietnamese Females With OTC Mutations
Source: Front Pediatr. 2020 Jul 23;8:321. doi: 10.3389/fped.2020.00321 (PMC7390877; doi:10.3389/fped.2020.00321)
Supplement: Supplementary file 1 [file Table_1.DOCX]

**Supplementary Table 1:** Mutation Taster analyses of the identified variants

| **Mutation** | **c.365A>T** | **c.717+1G>A** |
| --- | --- | --- |
| **Prediction** | **Disease causing** | **Disease causing** |
| **Summary** | **amino acid sequence changed.**  **known disease mutation at this position of amino acid (HGMD CM032299)** | known disease mutation at this position (HGMD CS003075)  known disease mutation: rs66500027 (pathogenic)  protein features (might be) affected  splice site changes |
| Alteration  (phys. location) | chr23:38240661A>T | chr23:38268049G>A |
| HGNC symbol | [OTC](http://www.genedistiller.org/cgi-bin/GeneDistiller_results.cgi?show_interaction=1&show_hpo=1&show_pathways=1&txid=9606&order=start_pos&show_generifs=1&show_mgd_phenotypes=1&show_GO=1&show_pfam=1&show_transcripts=1&show_omim=1&show_synonyms=1&show_interpro=1&show_paralogs=1&show_proteinfamilies=1&genesymbol=OTC) | [OTC](http://www.genedistiller.org/cgi-bin/GeneDistiller_results.cgi?show_interaction=1&show_hpo=1&show_pathways=1&txid=9606&order=start_pos&show_generifs=1&show_mgd_phenotypes=1&show_GO=1&show_pfam=1&show_transcripts=1&show_omim=1&show_synonyms=1&show_interpro=1&show_paralogs=1&show_proteinfamilies=1&genesymbol=OTC) |
| Ensembl transcript ID | [ENST00000039007](http://grch37.ensembl.org/Homo_sapiens/transview?db=core;transcript=ENST00000039007) | [ENST00000039007](http://grch37.ensembl.org/Homo_sapiens/transview?db=core;transcript=ENST00000039007) |
| Genbank transcript ID | [NM_000531](http://www.ncbi.nlm.nih.gov/nuccore/NM_000531) | [NM_000531](http://www.ncbi.nlm.nih.gov/nuccore/NM_000531) |
| Alteration type | single base exchange | single base exchange |
| Alteration region | CDS | intron |
| DNA changes | c.365A>T cDNA.517A>T g.28864A>T | g.56252G>A |
| AA changes | E122V Score: 121 | n/a |
| Position(s) of altered AA | 122 | n/a |
| Frameshift | no | n/a |
| Known variant | Variant was neither found in ExAC nor 1000G. known disease mutation at this position of amino acid,  (HGMD ID CM032299) | Allele 'A' was neither found in [ExAC](http://exac.broadinstitute.org/variant/X-38268049-G-A) nor 1000G.  known disease mutation: rs66500027  known disease mutation at this position (HGMD CS003075) |
| Regulatory features | H3K27me3, Histone, Histone 3 Lysine 27 Tri-Methylation | H3K27me3, Histone, Histone 3 Lysine 27 Tri-Methylation |
| phyloP / phastCons | \|  \| PhyloP \| PhastCons \| \| --- \| --- \| --- \| \| (flanking) \| 3.856 \| 1 \| \|  \| 3.175 \| 1 \| \| (flanking) \| 0.541 \| 1 \| | \|  \| PhyloP \| PhastCons \| \| --- \| --- \| --- \| \| (flanking) \| 2.873 \| 1 \| \|  \| 3.827 \| 1 \| \| (flanking) \| 3.072 \| 1 \| |
| Splice sites | no abrogation of potential splice sites | alteration within used splice site, likely to disturb normal splicing   \| Effect \| gDNA position \| score \| detection sequence \| exon-intron border \| \| --- \| --- \| --- \| --- \| --- \| \| Donor lost \| 56250 \| sequence motif lost \| - \| wt: AGAG\|gtat  mu: AGAG.atat \| \| Donor increased \| 56248 \| wt: 0.56 / mu: 0.98 \| wt: GCCAAAGAGGTATGC mu: GCCAAAGAGATATGC \|  \| |
| Distance from splice site | 22 | 2 |
| Protein features | no protein features affected | \| start (aa) \| end (aa) \| feature \| details \| \| --- \| --- \| --- \| --- \| \| 227 \| 240 \| Helix \| Might get lost \| \| 238 \| 238 \| \| Mod_Res \| N6-Acetyllysine \| \| --- \| --- \| \| Might get lost \| \| 244 \| 248 \| Strand \| Might get lost \| \| 250 \| 254 \| Helix \| Might get lost \| \| 258 \| 262 \| Strand \| Might get lost \| \| 263 \| 267 \| \| Region \| Ornithine Binding \| \| --- \| --- \| \| Might get lost \| \| 271 \| 273 \| Helix \| Might get lost \| \| 274 \| 280 \| Helix \| Might get lost \| \| 281 \| 283 \| Turn \| Might get lost \| \| 288 \| 292 \| Helix \| Might get lost \| \| 299 \| 302 \| Strand \| Might get lost \| \| 302 \| 305 \| \| Region \| Ornithine Binding \| \| --- \| --- \| \| Might get lost \| \| 303 \| 303 \| Act_Site \| Might get lost \| \| 308 \| 310 \| Turn \| Might get lost \| \| 313 \| 316 \| Helix \| Might get lost \| \| 323 \| 342 \| Helix \| Might get lost \| \| 330 \| 330 \| \| Binding \| Carbamoyl Phosphate. \| \| --- \| --- \| \| Might get lost \| \| 330 \| 330 \| \| Binding \| Ornithine. \| \| --- \| --- \| \| Might get lost \| |
| Length of protein | normal | n/a |
| AA sequence altered | yes | n/a |
| Position of stop codon in wt / mu CDS | 1065 / 1065 | 1065 / 1065 |
| Position (AA) of stopcodon in wt / mu AA sequence | 355 / 355 | 355 / 355 |
| Position of stopcodon in wt / mu cDNA | 1217 / 1217 | 1217 / 1217 |
| Position of start ATG in wt / mu cDNA | 153 / 153 | 153 / 153 |
| Chromosome | 23 | 23 |
| Strand | 1 | 1 |
| Last intron/exon boundary | 1158 | 1158 |
| Theoretical NMD boundary in CDS | 955 | 955 |
| Length of CDS | 1065 | 1065 |
| Coding sequence (CDS) position | 365 | n/a |
| DNA position | 517 | n/a |
| gDNA position | 28864 | 56252 |
| Chromosomal position | 38240661 | 38268049 |
