# Supplementary material for: Late-Onset Ornithine Transcarbamylase Deficiency and Variable Phenotypes in Vietnamese Females With OTC Mutations
Source: Front Pediatr. 2020 Jul 23;8:321. doi: 10.3389/fped.2020.00321 (PMC7390877; doi:10.3389/fped.2020.00321)
Supplement: Supplementary file 2 [file Table_2.DOCX]

**Supplementary Table 2:** Classification of the identified variants according to American College of Medical Genetics and Genomic recommendations

| **c.365A>T (p.Glu122Val): likely pathogenic variant** | | |
| --- | --- | --- |
| 2 Moderates | PM2 | Absent in 1,000 Genomes Project, Exome Variant Project, Genome Aggregation Database |
|  | PM5 | Novel missense change at an amino residue where a different missense change determined to be pathogenic has been seen before  This study: Glu122Val; previous study: Glu122Gly |
| 4 Supports | PP1 | Cosegregation with disease |
|  | PP2 | Missense variant in a gene that has low rate of benign missense variation and in which missense variants are a common mechanism of disease |
|  | PP3 | + SIFT, PolyPhen-2, Mutation Taster predicted disease causing.  + A glutamate residue at the position 122 was complete conservative among distinct species |
|  | PP4 | Patient’s phenotype or family history is highly specific for a disease with a single genetic etiology |
| **c.717+1G>A (IVS7+1G>A): pathogenic variant** | | |
| 1 Very strong | PVS1 | Null variant disrupting the canonical +1 splice sites at the end of exon 7 |
| 1 Moderate | PM2 | Absent in 1,000 Genomes Project, Exome Variant Project, Genome Aggregation Database |
| 4 Supports | PP1 | Cosegregation with disease |
|  | PP3 | MaxEnt and Human Splicing Finder predicted to break the donor site |
|  | PP4 | Patient’s phenotype or family history is highly specific for a disease with a single genetic etiology |
|  | PP5 | Reported as a pathogenic variant in ClinVar |
