# Supplementary material for: Late-Onset Ornithine Transcarbamylase Deficiency and Variable Phenotypes in Vietnamese Females With OTC Mutations
Source: Front Pediatr. 2020 Jul 23;8:321. doi: 10.3389/fped.2020.00321 (PMC7390877; doi:10.3389/fped.2020.00321)
Supplement: Supplementary file 3 [file Table_3.DOCX]

**Supplementary Table 3.** The missense mutations have detected in exon 4 in the *OTC* gene

| **No.** | **Genotype** | **Age of onset/**  **Gender** | **Phenotype** | **Reference** |
| --- | --- | --- | --- | --- |
| 1 | GGC→GAC  (Gly100Asp) | Late  Female | Reduced enzyme activity, hyperammonemia and orotic aciduria, positive allopurinol loading test | (1) |
| 2 | GCA→GAA  (Ala102Glu) | Neonatal  Male | - | (2) |
| 3 | GGA→AGA  (Gly106Arg) | Late  Female | Reduced enzyme activity | (3) |
| 4 | CTT→CCT  (Leu111Pro) | Late  Male | Severe phenotypes | (4) |
| 5 | CAT→CTT  (His117Leu) | Late  Male | Reduced enzyme activity  Milder disease | (5) |
| 6 | CAT→CTT  (His117Arg) | Late  Male | - | (6) |
| 7 | *GAA→GTA*  *(Glu122Val)* | *Late*  *Female* | *Mechanical ventilation, shock condition, deep coma, both of dilated pupils 4 mm with weak light reaction.* | *This study* |
| 8 | GAA→GGA  (Glu122Gly) | Late  Male | Elevated orotate excretion  Reduced enzyme activity | (7) (8) |
| 9 | ACG→ATG  (Thr125Met) | Neonatal  Male | OTC liver activity <1% | (9) |
| 10 | GAC→GGC  (Asp126Gly) | Neonatal  Male | OTC activity:0.9% | (10) |
| 11 | CGT→CAT  (Arg129His) | Late  Male & Female | Psychiatric and behavioral symptoms, mild hyperammonemia, consistently elevated plasma glutamine, orotic aciduria | (11) |
| 12 | CGT→CTT  (Arg129Leu) | Late  Male & Female | Hyperammonemia and raised levels of methionine, alanine and glutamine  OCT activity: 5.5-20%  Retarded, having little vocabulary and limited social skills | (12) |

**References**

1. Leibundgut EO, Liechti‐Gallati S, Colombo J-P, Wermuth B. Ornithine transcarbamylase deficiency: Ten new mutations and high proportion of de novo mutations in heterozygous females. *Human Mutation* (1997) **9**:409–411. doi:10.1002/(SICI)1098-1004(1997)9:5<409::AID-HUMU5>3.0.CO;2-Z

2. Tuchman M, Morizono H, Rajagopal BS, Plante RJ, Allewell NM. Identification of “private” mutations in patients with ornithine transcarbamylase deficiency. *J Inherit Metab Dis* (1997) **20**:525–527. doi:10.1023/a:1005301513465

3. McCullough BA, Yudkoff M, Batshaw ML, Wilson JM, Raper SE, Tuchman M. Genotype spectrum of ornithine transcarbamylase deficiency: Correlation with the clinical and biochemical phenotype. *Am J Med Genet* (2000) **93**:313–319. doi:10.1002/1096-8628(20000814)93:4<313::AID-AJMG11>3.0.CO;2-M

4. Grompe M, Caskey CT, Fenwick RG. Improved molecular diagnostics for ornithine transcarbamylase deficiency. *Am J Hum Genet* (1991) **48**:212–222. Available at: https://www.ncbi.nlm.nih.gov/pmc/articles/PMC1683033/ [Accessed November 28, 2019]

5. Tuchman M, Plante RJ, Giguère Y, Lemieux B. The ornithine transcarbamylase gene: new “private” mutations in four patients and study of a polymorphism. *Hum Mutat* (1994) **3**:318–320. doi:10.1002/humu.1380030325

6. Matsuda I, Tanase S. The ornithine transcarbamylase (OTC) gene: Mutations in 50 Japanese families with OTC deficiency. *Am J Med Genet* (1997) **71**:378–383. doi:10.1002/(SICI)1096-8628(19970905)71:4<378::AID-AJMG2>3.0.CO;2-Q

7. Arranz JA, Riudor E, Marco-Marín C, Rubio V. Estimation of the total number of disease-causing mutations in ornithine transcarbamylase (OTC) deficiency. Value of the OTC structure in predicting a mutation pathogenic potential. *J Inherit Metab Dis* (2007) **30**:217–226. doi:10.1007/s10545-007-0429-x

8. Gao H, Li W, Yan Z, Jiang M, Rui D, He Y. Molecular characterization of a new mutation E122G of human ornithine transcarbamylase gene. *Zhonghua Yi Xue Yi Chuan Xue Za Zhi* (2003) **20**:19–22.

9. Gilbert-Dussardier B, Segues B, Rozet JM, Rabier D, Calvas P, de Lumley L, Bonnefond JP, Munnich A. Partial duplication [dup. TCAC (178)] and novel point mutations (T125M, G188R, A209V, and H302L) of the ornithine transcarbamylase gene in congenital hyperammonemia. *Hum Mutat* (1996) **8**:74–76. doi:10.1002/(SICI)1098-1004(1996)8:1<74::AID-HUMU11>3.0.CO;2-O

10. Matsuura T, Hoshide R, Kiwaki K, Komaki S, Koike E, Endo F, Oyanagi K, Suzuki Y, Kato I, Ishikawa K. Four newly identified ornithine transcarbamylase (OTC) mutations (D126G, R129H, I172M and W332X) in Japanese male patients with early-onset OTC deficiency. *Hum Mutat* (1994) **3**:402–406. doi:10.1002/humu.1380030415

11. Tuchman M, Plante RJ, McCann MT, Qureshi AA. Seven new mutations in the human ornithine transcarbamylase gene. *Hum Mutat* (1994) **4**:57–60. doi:10.1002/humu.1380040109

12. Strautnieks S, Malcolm S. Novel mutation affecting a splice site in exon 4 of the ornithine carbamoyl transferase gene. *Hum Mol Genet* (1993) **2**:1963–1964. doi:10.1093/hmg/2.11.1963
